# Proceedings of the American Medical Association

**Published:** 1854-06

**Authors:** 


### PROCEEDINGS OF THE AMERICAN MEDICAL ASSOCIATION,

*At the Annual Session held in St. Louis, Missouri, May*, 1854.

Pursuant to the resolution adopted at the last Annual Session, the members of the American Medical Association assembled at St. Louis, Mo., on Tuesday, 2d May, 1854. At eleven o'clock, A. M., the Association was called to order in the Verandah Hall, by Dr. USHER PARSONS, of Rhode Island, the senior Vice-President of the Association, and the following letter was read from Dr. JONATHAN KNIGHT, of Connecticut, the President of the Association, in which he announced his inability to be present :

*New Haven, Conn., April* 25, 1854.

To Edwin S. Lemoine, M. D., Secretary of the American Medical Association, St. Louis, Mo.

*Dear Sir*,—As the time is near at hand for the assembling of the American Medical Association, it is proper for me to inform you, and through you the members of the Association, that it will not be in my power to be present at the annual meeting. I have come to this conclusion after much reflection, with great reluctance. The meetings of the Association have always been periods of high gratification to me.

The acquaintance there formed, and the intercourse had with the members of the profession from every part of the country, have been among the most gratifying events of my life. I have looked forward to the annual meeting of this year with the anticipation of unusual satisfaction. There was a portion of the country new to me to be visited ; there were members of the profession, probably in large numbers, few of whom were known to me, to become acquainted with, in addition to the ordinary attractions of the meeting, which made me strongly desirous for my own sake to be present.

At the same time, I feel the full weight of the obligation to attend the meeting this year, arising from the high honor which has been conferred upon me by the Association. Perhaps no one so little deserving has received so many and so great favors from the medical profession, and I take this opportunity to renew my acknowledgments for these favors.

It is with no ordinary emotion that, although absent, I greet those of my professional brethren who will be present at St. Louis. I do this with the most cordial feelings, and with the strongest wishes for their welfare. Allow me, also, to express the confident hope that their wise deliberations will result in the promotion of the best interests of the profession, and advantage of the country, and to ask that the blessing of God may rest upon their labors.

With esteem and respect, your ob't servant, J. KNIGHT.

The following letter was next read from Dr. KNIGHT to Dr. PARSONS, notifying him of his (Dr. Knight's) intended absence :

*New Haven, April* 15*th*, 1854.

To Usher Parsons, M. D., Providence.

*My Dear Sir*,—As the time is near at hand for the meeting of the American Medical Association at St. Louis, it is proper for me to inform you that I shall not probably be able to attend it. Matters of a purely personal nature will, I suppose, deprive me of that pleasure. As you are the Senior Vice President of the Association, I trust you will be present and perform the duties which will devolve upon you. I give you this early notice that you may have time to prepare such an address as may be proper for the occasion. That you will do this as you do everything else, (in the best manner,) I have no doubt. With esteem and respect, your obedient servant, J. KNIGHT.

Dr. E. S. LEMOINE, of Missouri, one of the Secretaries, next read a letter from Dr. E. S. BEADLE, of N. Y., in which he expressed his inability to attend and discharge the duties of one of the Secretaries.

Dr. JAMES R. WASHINGTON, of St. Louis, Mo., Chairman of the Committee of Arrangements, now welcomed the assembled delegates, on behalf of the profession of St. Louis, in a most cordial and eloquent address.

The Vice-President, Dr. PARSONS, announced that the Association was now formally organized, and that the first business in order would be the reading of the credentials of the delegates.

The roll was accordingly read by States and Societies, and the following delegates present answered to their names :

*Maine* —Joseph H. Eastbrook, M. D., Maine Medical Association ; Charles Millet, M. D., do. ; Sylvester Oakes, M. D., Lewiston Falls Medical Association.

*Massachusetts.*—Dr. John Green, Masachusetts Medical Society; Dr. A. A. Gould, do. ; Dr. John Flint, Boston Society for Medical Improvement ; L. N. Bates, M. D., Worcester Co. Medical Society ; D. H. Storer, M. D., Mass. Gen'l Hospital ; Ephraim Lovell, M. D., do. ; Benj. F. Haywood, M. D., do. ; Alfred Hitchcock, M. D., do.

*Rhode Island.*—Dr. Usher Parsons.

*Connecticut.*—Dr. Nathan R. Ives, Con't Medical Society ; Dr. L. G. Rockwell, do. ; Dr. Charles Hooker, Medical Institute of Yale College.

*New York.*—S. K. French, M. D., Medical Association of Central New York ; Joel E. Hawley, M. D., Geneva Medical College ; James D. Phelps, M. D., New York County Medical Society ; Thos. W. Blatchford, M. D., New York State Medical Society ; Caleb Budlong, M. D., Herkimer County Medical Society ; R. D. Allen, M. D., New York State Medical Society ; Henry S. Douns, M. D., N. Y. County Medical Society ; Lewis A. Sayre, M. D., N. Y. Academy of Medicine ; Alden March, M. D., Albany Medical College ; Chandler R. Gillman, M. D., N. Y. College of Physicians and Surgeons ; E. H. Davis, M. D., N. Y. Medical College ; Jas. P. White, M. D., University of Buffalo.

*New Jersey.*—G. R. Chetwood, M. D., Essex Dist. Med. Soc'y ; L. A. Smith, M. D., N. J. State Medical Society.

*Pennsylvania.*—John L. Atlee, M. D., and Jno. C. Ross, M. D. State Medical Society ; Réné La Roche, M. D., Philadelphia County Medical Society ; John B. Biddle, M. D., College of Physicians, of Philadelphia ; Samuel Keannegy, M. D., John Kean, M. D., Samuel Parker, M. D., Isaac C. Weiddler, M. D., Lancaster County Medical

Society; Isaac Thomas, M. D., Chester County Medical Society; N. L. Hatfield, M. D., N. Med. Association of Philadelphia; George W. Norris, M. D., Pennsylvania Hospital; Francis West, M. D., Philad'a Association for Med. Instruction; Joseph Carson, M. D., and Joseph Leidy, M. D., University of Pennsylvania; John B. Biddle, M. D., Medical Department of Pennsylvania College; Samuel H. Meade, M. D., and J. B. Bell, M. D., Medico-Chirurgical College; Wm. Keith, M. D., Permanent Member.

*Virginia.*—Adam Spitler, M. D., Medical Society of Virginia.

*South Carolina.*—J. L. Dawson, Medical Society of South Carolina; Robt. S. Bailey, S. C. Medical Association; Wm. T. Wragg, Medical Society of S. C.; Henry R. Frost, Faculty Medical College of S. C.; Thos. G. Prioleau, do.; Wm. H. Ford, Medical Society of S. C.; Wm. C. Ravenel, S. C. Medical Association; J. Ford Prioleau, Medical Society of S. C.

*Alabama.*—S. W. Clanton, Alabama State Medical Association.

*Mississippi.*—T. J. Grafton, Jefferson Medical Association.

*Louisiana.*—E. D. Fenner, La. State Medical Society.

*Cherokee.*—R. D. Ross, Medical Society of Cherokee Nation.

*Kentucky.*—John R. Allen, Medical Department Transylvania University; Saml. D. Gross, University of Louisville; R. J. Breckenridge, Kentucky Medical School; J. W. Scott, U. S. Marine Hospital, Louisville; John Magoffin, do.; Walter A. Norwood, Permanent Member.

*Ohio.*—W. W. Taggart, Wayne co. Medical Society; J. D. Robinson, do.; W. T. Taliaferro, Cincinnati Medical Society; R. D. Mussey, Miami Medical College; Robt. R. McIlvaine, Medico-Chir. Society of Cincinnati; O. M. Langdon, do.; J. J. Arons, do.; Wm. Clendenin, do.; Amos C. Smith, State Medical Association of Ohio; J. Y. Updergraff, Belmont Medical Society; S. M. Smith, Starling Medical College; C. B. Hughes, Medico-Chir. Society, Cincinnati; J. E. Nagle, Hardin co. Medical Society; Geo. Mendenhall, Miami Medical College; J. A. Coons, Montgomery co. Medical Society; Joshua Clements, do.

*Indiana.*—Wm. H. Byford, Evansville Medical College; D. Morgan, Evansville Medical Society; W. W. Hitt, Vincennes Medical Society.

*Missouri.*—Wm. M. McPheeters, Medical Department St. Louis University; R. S. Holmes, do.; Jas. R. Washington, St. Louis Medical Society; S. Gratz Moses, do.; A. J. Coons, do.; E. S. Lemoine, do.; F. E. Baumgartner, do.; Geo. S. Walker, do.; W. S. Edgar, do.; W. S. Golding, do.; Chas. W. Stevens, O'Fallon Dispensary; Alfred Behr, German Medical Society of St. Louis; Adolphus Wislizenus, do.; M. L. Linton, St. Louis City Hospital; C. A. Pope, do.; E. Y. Bannister, United States City Hospital; J. B. Johnson, do.; George Johnson, United States Marine Hospital; John C. Wellorn, Pike County Medical Society; George Engleman, Medical Association of the State of Missouri; Thos. Reyburn, do.; Daniel Sandy, do.; John Barnes, do.; J. Pollak, do.; L. P. Perry, do.; H. A. Prout, do.; David M. Cooper, do.; S. W. Adreon, do.; Alexander Marshall, do.; J. W. Wilson, do.; H. Schoenich, do.; Wm. P. Boulware, do.; Isaac P. Vaughan, do.;

Hammond Schoemaker, do; John Laughton, do.; W. A. Jenkins, do.; John S. Moore, Medical Department of the University of Missouri; Jos. M. McDowell, do.; John B. Alexander, Lafayette County Medical Association; J. B. Atkinson, do.; Charles Quarles Chandler, Missouri State Medical Society; Wm. Webb, Permanent Member; J. S. B. Alleyne, St. Louis Medical Institute; J. O. F. Farrar, do.; Abner Hopton, Dispensary of the University of Missouri; Charles W. Hempstead, Permanent Member; Richard G. Barrett, St. Louis County Hospital; Geo. Penn, do.; W. A. Curry, State Prison Hospital; R. K. Lewis, Randolph County Medical Society; John M. McKeage, St. Louis County Lying-in Hospital; Jos. Buron, St. Louis County Insane Hospital; J. W. B. Reynolds, Franklin County Medical Society; Pierce M. Butler, do.; Charles L. Boisliniere, Biddle Lying-in Hospital; M. M. Pallen, do.; F. P. Leavenworth, St. Louis Quarantine Hospital; A. Litton, Permanent Member.

*Michigan.*—A. B. Palmer, State Medical Society; J. H. Beech, do; Wm. Brodie, Detroit Medical Society; Andrew Murray, Medical Association of Michigan.

*Iowa.*—John D. Elbert, State Medical Society; Thomas Silvester, do.; John F. Fly, North Western Medical Society; L. McGuigan, Iowa University Medical Department; E. R. Ford, do.; E. A. Arnold, City Hospital, Keokuk; N. Van Patten, Clinton Medical Society; J. L. Sanford.

*Illinois.*—E. S. Cooper, Illinois State Medical Society; Samuel Thompson, do.; Thomas D. Washburn, Æsculapian Medical Society; James Bunce, Knox county Medical Society; J. W. Spalding, do.; Wm. Wood, Alexandria county, Illinois; George W. Stipp, McLane Medical Society; Y. P. Rogers, do.; S. Y. Baldwin, Mason Medical Society; S. T. Trowbridge, do.; E. L. Colburn, Peoria Medical Society; M. Shepherd, member; N. S. Davis, Hospital of the Sisters of Mercy, Chicago; Daniel Brainard, Rush Medical College; W. B. Herrick, do.; J. S. Maus, Pekin Medical Society; J. C. Hinsey, do.; Joseph Stout, Lasalle county Medical Society; George T. Allen, Madison county Medical Society; Rudolphus Rouse, State Medical Society, and Peoria Medical and Surgical Society; H. A. Johnson, State Medical Society; Wm. W. Welsh, do.; Samuel Long, do.; James Bloodgood, Cook county Medical Society; Adam Nichas, permanent member, Quincy; Charles W. Clarke, Winnebago county Medical Society; Daniel Stahl, Adams county Medical Society; W. C. Quigley, Cook county Medical Society; J. N. Ralston, Adams county Medical Society; W. H. Davis, Medical Society, Illinois.

*Wisconsin.*—J. B. Dousman, State Medical Society; J. K. Bartlett, do.; John H. Murphy, Minesota Medical Society.

*Tennessee.*—Lewis Shanks, Memphis Medical College, A. P. Merrill, do.; E. R. Dabney, Montgomery county Medical Society; J. L. C. Johnstone, do.; Frank A. Ramsey, Tennessee Medical Society; Wm. D. Haggard, Sumner county Medical Society; H. M. Clements, Medical Society, Tennessee; J. B. Lindsley, do.; Wm. K. Boling, Medical

Dept. of the University of Nashville; Paul F. Eve, University of Nashville; C. B. Guthrie, Permanent Member.

*U. S. Navy.*—Ninian Pinkney, M. D.

After the calling of the Roll, the Report of the Committee of Publication was read, and, on motion of Dr. ATLEE, of Pennsylvania, was laid on the table for the present.

On motion of Dr. J. P. WHITE, of Buffalo, N. York, a recess of fifteen minutes was now taken, to allow the State Delegations respectively to meet and appoint representatives in the Nominating Committee to report Officers for the Association.

After the recess, the various State Delegations reported the following gentlemen, as the Nominating Committee :—

*Maine.*—Dr. Charles Millett.
*Massachusetts.*—Dr. D. H. Storer.
*Connecticut.*—Dr. P. G. Rockwell.
*New York.*—Dr. J. P. White.
*New Jersey.*—Dr. George R. Chitwood.
*Pennsylvania.*—Dr. Rene La Roche.
*Virginia.*—Dr. Adam Spitler.
*Minnesota.*—Dr. J. H. Murphy.
*South Carolina.*—Dr. Thomas G. Prioleau.
*Illinois.*—Dr. W. B. Herrick.
*Alabama.*—Dr. S. W. Clanton.
*Louisiana.*—Dr. E. D. Fenner.
*Missouri.*—Dr. Thomas Reyburn.
*Michigan.*—Dr. William Brodie.
*Mississippi.*—Dr. T. J. Grafton.
*Iowa.*—Dr. T. Seviter.
*Tennessee.*—Dr. J. B. Lindsley.
*Wisconsin.*—Dr. J. B. Dousman.
*Kentucky.*—Dr. Robert J. Breckenridge.
*Ohio.*—Dr. O. M. Langdon.
*Indiana.*—Dr. W. W. Hitt.

On motion of Dr. ATLEE, it was resolved, that the morning Sessions of the Association shall be from 9 A. M. to 1 P. M., and the afternoon Sessions from 3 to 5 P. M.

Dr. BRAINARD, of Chicago, Illinois, offered the following resolution: That the future meetings of the Association shall be held alternately in the Eastern, Southern, and Western Sections of the Union.

After some discussion, on motion of Dr. BARNES, of St. Louis, this resolution was laid on the table.

Dr. BIDDLE, of Pennsylvania, on behalf of the Philadelphia delegation, tendered an invitation from the profession of Philadelphia to the Association, to hold its next annual meeting at Philadelphia.

On motion, the Association adjourned to 3 P. M.

*Afternoon Session.*

Pursuant to adjournment, the Association met at 3 P. M.

The Acting President, Dr. PARSONS, read the following Annual Address, the preparation of which had devolved on him, in consequence of the absence of the President, Dr. Knight.

*Gentlemen of the American Association:*

It has been customary for the presiding officer of this Association, on retiring from the Chair, to give a valedictory discourse. On the eve of my departure from Rhode Island our venerable President notified me of his inability to attend and perform this part of his official duty, which deprives us of the rich entertainment anticipated from so distinguished a scholar and professor. The notice being entirely unexpected, I am unprepared to offer you anything worthy of your attention, and my inclination would, therefore, be to remain silent, but for an apprehension that this course might operate as a precedent to others, on similar occasions. I will, therefore, present you, rather as an apology for a discourse, a few thoughts that have suggested themselves while on my way to this city.

In order to promote the honor, dignity and usefulness of our profession, objects for which the Association was instituted, its members must be gathered from all parts of our country and united into one harmonious fraternity, and must adopt such measures as will promote and perpetuate among ourselves an *esprit de corps*, a conformity of sentiment and feeling, and a combination and co-operation in action. This has already been accomplished in a degree by holding our annual meetings in distant and remote cities of the Union. They must continue to be carried to new and ever-varying spheres of action, until their beneficial influence is made available to the whole profession. As the metallurgist, in separating a heterogeneous mass of particles, passes over it a magnetic bar, to attract the pure iron and steal with a force proportioned to its proximity, so must the meetings of this Association, in order to gather into one fold suitable materials of growth and strength, be carried from place to place over the wide mass of our population, attracting from the dross and impurities all that is valuable and worthy of reception and incorporation into a homogeneous and efficient brotherhood. These considerations influenced me in voting to accept the invitation to hold the present meeting in Missouri, notwithstanding the toil and fatigue it imposed upon a large proportion of the delegates. It is here, more than elsewhere, that the meetings of the Association are likely to prove beneficial, by a rapid enlargement of our numbers.

Whoever glances at a map of the Mississippi Valley, extending from the base of the Alleghany and Cumberland Mountains to the margin of the Rocky Mountains—from the highlands bordering on Lake Superior to the Gulf of Mexico—and contemplates the fertility of its soil, its adaptation for cereal productions, which are so necessary for human subsistence and increase, and who surveys the majestic Mississippi, navigable through this whole territory, with its numerous navigable tributaries pouring in their treasures on either side, and adds to this the vast mineral resources, lead, copper, iron and coal, which are far more conducive to healthful opulence than the golden regions of California; whoever, I say, cordially surveys all these elements of future growth, expansion and power, and moved onward by the agency of steam, on land and water, and in labor-saving mechanical and manufacturing operations, can arrive at no other conclusion than that this vast territory, the largest and most favored one by nature, of any under the whole canopy of Heaven, will in time be densely populated *with scores of millions,* and become the seat of empire of the western world, and that it is destined to be the grand theatre of human progress, in every department that is calculated to advance the dignity and promote the happiness of the human family.

And in no department of human affairs is progress here more sure than in medical knowledge. Our Atlantic States have inherited a reverence for European opinions, which, although commendable in our early medical history, is, at the present day, less favorable to American progress and discovery in medicine. We need to interrogate nature and experience more, and European opinions less; we need mental as well as political independence—a freer swing of thought and purpose that characterizes our brethren of the West, and which this Association is adapted to call into action.

There is much to encourage you in your recent discoveries and contributions—in the results of vivisections of saurians, the half of which, if confirmed by future experiments, will shed new light on Physiology. And again, in the discoveries made relating to the process of digestion, by your late lamented BEAUMONT, of St. Louis, who, for theories and speculations before prevailing, has substituted ocular demonstration of the modus operandi of that wonderful process, by submitting to it the various articles of human aliment, and determining the length of time required for converting each into healthful chyme: and again in the successful labors of DRAKE, in travelling from State to State throughout the valley, collecting the history and character of its epidemics, by personal inquiry and observation. Others of your venerated dead might be mentioned, who have pursued a like independent course untrammeled by prevailing European authorities. Of their immediate successors, who now stand at the head of the profession, it would ill become me to speak, seeing that some of them are present and unused to such freedom of remark. But to the junior members of the profession we would say, unite with us—follow the example of the distinguished pioneers I have named, and of Caldwell and Harrison who have gone to their reward—throw the result of your labors into the common stock of medical knowledge accumulated by this association, where, rest assured, they will be duly appreciated and be appropriated to the common benefit of the profession and of mankind, and redound eventually to your everlasting honor and professional fame.

Gentlemen, eight years have elapsed since the preliminary meeting of the convention which recommended the formation of this National Association, and the results of its labors have equalled the expectations of the friends of reform and progress in our profession. The six published volumes of transactions have successively increased in value and interest, and are enduring monuments of the ardent zeal and patient industry of the numerous contributors, and there is every reason to hope that our future labors will continue to be crowned with equally increasing value and interest.

Gentlemen, we are reminded by the history of the past year of the frailty and uncertainty of human life. Death has removed many of our brethren of this association, and among them the illustrious BEAUMONT, already mentioned, and Dr. GEORGE C. SHATTUCK, LL. D., of Boston, an extensive and highly esteemed practitioner, and formerly President of the Massachusetts Medical Society.

He was reputed the wealthiest physician in New England, and his numerous bequests to educational, humane and religious iustitutions and enterprises, and to private charities, proclaim that his philanthropy was proportioned to his opulence.

We are reminded, by the return of this anniversary, of the terrible catastrophe that occurred at Norwalk. The Association had received from our brethren of New York a cordial welcome, and were honored with overflowing hospitality. After a delightful and profitable session, the Association adjourned, and many of them were on their way in cars to their respective homes, in joyous anticipation of rejoining their families, when in a moment—in the twinkling of an eye—seven of them were launched into eternity, leaving us the solemn admonition that in the midst of life we are in death. I should deem it a duty, on this occasion, to pay a tribute of respect to their memory, by portraying their many virtues and excellencies, as men and as physicians, had I not learned that justice will be done them by an abler pen. Our brethren of New York, with characteristic magnanimity, which adds to their claims on our gratitude, immediately on the announcement of the disaster, summoned a meeting, and passed resolutions expressive of their deep sorrow at the sad event, and tendering their condolence to the bereaved families. They also appointed a committee to prepare an eulogy on the deceased, to be offered at this annual meeting; and the distinguished ability of the chairman and members of that committee is a sufficient guarantee that justice will be done to the memory of these our lamented brethren.

A resolution was now offered that the address be referred to the Committee of Publication for incorporation in the transactions of the Associations, which was carried.

A letter was read, announcing that the sixth volume of the Transactions had been presented to the Imperial Academy of Medicine at Paris.

The following memorial by members of the Medical Association of the city of New York, was now read:

*To the American Medical Association.*

At a special meeting held in the city of New York, on the 12th of May, 1853, of such members "of the American Medical Asssociation as reside in the city and its vicinity, and such as were remaining here from abroad, for the purpose of expressing their feelings respecting the disaster on the New York and New Haven Railroad at Norwalk, in Connecticut, which resulted in the death of so many valuable members of the Association," after adopting sundry resolutions expressive of their sentiments and sympathy with the bereaved, a committee of seven was appointed to devise some suitable method of commemorating the event, and the worth and professional character of our lamented associates, and to recommend said plan to the next annual meeting of the Association.

At a meeting of the Committee thus appointed it was resolved, that, in the opinion of the Committee, the most appropriate method of carrying into effect the objects had in view in their appointment, would be by preparing a narrative of the event, together with a brief biographical sketch of each individual, which shall embrace a notice of the birth-place, age, place of education, when and where they derived their medical authority, where located after entering the profession, tastes and habits of life, if any, to what particular branch of the profession devoted, what positions held in the profession, either as professors, presidents or officers of Medical Societies, what literary labors, medical or otherwise, performed, what done to advance the science of Medicine; and that such narrative and biographical memoirs be published in the next volume of of the Transactions of the Association.

The Chairman and Secretary of the Committee beg leave to state that, although they have taken measures to procure the materials for preparing the Biographical Memoirs, answers to all the letters of inquiry have not been received. In reporting the above proceedings of the Committee to the Association, they would respectfully recommend the adoption of the plan proposed, and suggest that they be authorized to complete the narrative and memoirs in question, and to transmit them to the Committee for publication. JOS. M. SMITH, M. D., Chairman.

To E. L. BEADLE, M. D., Secretary.

*New York, April 24th,* 1854.

The following resolutions passed at the annual meeting of the New Hampshire Medical Society were read:

At the Annual Meeting of the New Hampshire Medical Society, held at Concord, June 1, 1853, the following Resolutions were unanimously adopted:

Resolved, That it is the decided opinion of the New Hampshire State Medical Society, that no Delegate should be admitted to membership in the American Medical Association, who represents a medical society which numbers among its members any person or persons who adopt as their system of practice any form of empiricism.

Resolved, That the Secretary of this Society be instructed to transmit a copy of this resolution to the Secretaries of each of the State Medical Societies, and to the Secretaries of the American Medical Association, previous to their next Annual Meeting. E. K. WEBSTER,

Secretary N. H. Medical Society

*Boscawen, June,* 1853.

Dr. GROSS, of Louisville, Ky., offered the following resolution: That hereafter it shall be disorderly for the profession in any city in which the Association may hereafter meet, to give costly entertainments.

This resolution was discussed by the mover, Dr. COONS, of St. Louis, and others, when a substitute, offered by Dr. MCPHEETERS, of St. Louis, and accepted by Dr. Gross, was adopted, to the effect that the Association discountenances for the future all extravagant public entertainments on the occasion of their meetings.

Dr. WHITE, from the Nominating Committee, made the following report, and the officers named therein were afterwards unanimously elected by the Association.

*For President.*—CHARLES A. POPE, M. D., of Missouri.
*Vice Presidents.*—E. D. FENNER, M. D.. of Louisiana.
" " N. S. DAVIS, M. D., of Illinois.
" " WM. T. WRAGG, M. D., of South Carolina.
" " JOHN GREEN, M. D. of Massachusetts.
*Secretaries.*—E. S. LEMOINE, M. D. of Missouri.
" FRANCIS WEST, M. D., of Pennsylvania.
*Treasurer.*—D. F. CONDIE, M. D., of Pennsylvania.

Dr. STORER, of Boston, Dr. WHITE, of Buffalo, Dr. BRAINARD, of Chicago, and Dr. REED, of Tennessee, were appointed a committee to conduct the newly appointed officers to their seats on the platform.

In the absence of Dr. POPE, who was not in attendance, owing to indisposition in his family, Dr. FENNER, the senior Vice-President, took the Chair.

On motion of Dr. PALMER, of Michigan, it was resolved that the next Annual Meeting of the Association be held at Philadelphia.

The resolutions, forwarded by the Chairman of the Committee of Publication, were now taken up. They fix the annual assessment upon the members at *three dollars*, and authorize the Treasurer to erase the names of all members who shall be a year in arrears.

On motion of Dr. BIDDLE, these resolutions were so amended as to require the Treasurer, before erasing the name of any member, to issue a circular informing him of his indebtedness; and, as thus amended, the resolutions were adopted.

Dr. BRODIE, of Michigan, under instructions from the Detroit Medical Society, read an invitation to the Association to hold its next Annual Meeting at Detroit. In view, however, of the selection of Philadelphia, just made, he moved that the resolution lie on the table. Carried.

DR. ATLEE, from the committee to prepare a stone for the Washington Monument, made a report, in which he described the stone and inscription prepared. The funds raised being yet insufficient to defray the expenses incurred, DR. ATLEE invited further contributions.

DR. CHARLES HOOKER, of Connecticut, was appointed Treasurer pro tempore, in the absence of the Treasurer elect.

It being now announced that DR. POPE, the President elect, was in the hall, he was conducted to the chair by the committee appointed for the purpose; and, upon assuming the duties of his office, Dr. POPE returned thanks in a few highly appropriate and eloquent remarks.

DR. PINKNEY, U. S. Navy, now by request addressed the Association, and returned the thanks of the medical officers of the Navy to the Association, for its sympathy and co-operation in the long struggle for their just rights of rank, in which the medical officers of the Navy had been engaged. That struggle, he believed, was near a termination; and the rank which was desired by his corps, would soon by law be conferred upon them, there was every reason to anticipate.

After the reading of several invitations, the Association adjourned.

*Second Day, Wednesday, 4th May.*

The meeting convened as per adjournment at nine o'clock, A. M., Dr. POPE, President, in the Chair.

The minutes of the preceding day were read, and after one or two slight amendments, were adopted.

The Chairman of the Committee of Arrangements reported the following Delegates as having registered their names since Tuesday:

Richard M. Cooper, Camden, New Jersey; F. S. Schenck, Somerset county, New Jersey; John A. French, Cheshire county, New Hampshire; Geo. R. Grant, Memphis, Tennessee; H. B. Musgrave, Cincinnati, Ohio; L. D. Waterman, Cincinnati, Ohio; M. B. Wright, Cincinnati, Ohio; J. C. Hughes, Keokuk, Iowa; Jas. W. Stone, Boston; John A. Ranch, Burlington, Iowa; David Prince, Jacksonville, Illinois; E. R. Dabney, Clarksville, Tennessee; J. L. C. Johnson, New Providence; S. A. Paddock, Princeton, Illinois; Wm. Corson, Norristown, Pennsylvania.

The names of several gentlemen were offered as members by invitation, and were accepted.

On motion, it was resolved, that the afternoon session be to-day adadjourned at 4 P. M.

On motion of Dr. ATLEE, a memorial from the American Medical Society of Paris to the A. M. Association, was read and referred to the Committee on Medical Education.

The President announced that the reading and consideration of the annual reports of committees would now be in order. The committees were accordingly called, as follows:

Dr. D. F. CONDIE, of Philadelphia, on the Causes of Tubercular Disease, was not prepared to report, and requested further time.

Dr. GEO. B. WOOD, of Philadelphia, on Diseases of Parasitic Origin not being present, had sent a verbal request to be discontinued. His request was accordingly granted.

Dr. JOHN L. ATLEE, of Lancaster, Pa., on Epidemics of New Jersey, Pennsylvania, Delaware and Maryland, not being prepared to make a full report, requested to be continued on the same committee.

Dr. D. J. CAIN, of Charleston, S. C., on Epidemics of South Carolina, Florida, Georgia and Alabama, read an abstract of his report. It was referred to the Committee on Publication.

Dr. W. L. SUTTON, of Georgetown, Ky., on Epidemics of Tennessee and Kentucky. He had made a partial report, but of such meagre materials that he requested to be continued. His report was referred to the Committee of Publication when ready.

Dr. GEORGE MENDENHALL, of Cincinnati, Ohio, on the Epidemics of Ohio, Indiana and Michigan. He presented a report for the year 1852 and 1853, of which he read a brief abstract. The report was referred to the Committee on Publication with the request to have it published in the proceedings of the present year.

Drs. PALMER and ATLEE each dwelt at some length upon the great and lasting good that might be accomplished if all the members of the Association would duly record their individual experience of epidemics, and report all such cases to the Chairmen of the Committees. Dr. ATLEE had been two years Chairman of such a committee, and during that time had only received two such reports,—one from New York, and a partial one from Pennsylvania. He stated that he had used great exertions to get professional men to co-operate in this work, and appealed to the whole profession to do everything in their power to promote this great object. He very ably urged the necessity of co-operation by all the profession with the several committees having these reports in charge.

Dr. R. S. HOLMES, of St. Louis, Mo., on Epidemic Erysipelas read an abstract of his report. It was referred to the Committee on Publication.

Dr. E. D. FENNER, of New Orleans, on Epidemics of Louisiana, Mississippi, Texas and Arkansas. He read a comprehensive abstract of his report—dwelling principally on the ravages of the cholera and yellow fever, and the causes and the means of treatment.

Dr. FENNER had not completed his report, and Dr. MCPHEETERS offered a resolution that Dr. FENNER be requested to complete his report, and submit it to the Committee on Publication to be published. The resolution was adopted.

Dr. MUSSEY, of Cincinnati, now made a motion to suspend the order of regular business, to allow Dr. LINTON, of St. Louis, to express his views with regard to the Pathology of the Yellow Fever. A suspension of business was made for the purpose.

The next report called for was that of Dr. DANIEL BRAINARD of Chicago, Illinois, on the Constitution and Local Treatment of Carcinoma. He requested further time to make a full report.

Dr. N. S. DAVIS of Chicago, Illinois, on the Influence of Local Circumstances on the Origin and Prevalence of Typhoid Fever. The report, of which he read a brief abstract, was referred to the Committee on Publication.

Dr. DONALDSON of Baltimore, on the Present and Prospective value of the Microscope in Disease. Dr. DONALDSON, in a communication, stated that his report was complete, as he was not being present, it was without reading, referred to the Committee on Publication.

The Report of the Committee on Medical Education being called for, it was stated that Dr. WELLFORD, of Virginia, the Chairman, had transferred the duty assigned him to Dr. CABELL, of Virginia. Dr. Cabell had prepared a report, but (as we understood) it had not been transmitted to the Association. It was referred to the Committee on Publication.

The Report of the Committee on Medical Literature, Dr. T. S. BELL, of Kentucky, Chairman, was called for, but no report was presented.

The Report of the Committee on Plans for the organization of State and County Societies, Dr. ISAAC HAYS, of Pennsylvania, Chairman, was called for, but no report was presented.

Dr. POPE, Chairman of the Committee on Prize Essays and Volunteer Communications, now made the following report:

Mr. PRESIDENT: The Committee on Prize Essays and Volunteer Communications, respectfully report that the Essays submitted to their consideration were nine in number, of which one was presented as a volunteer communication. The committee have carefully examined the whole of these Essays and bestowed upon them the attention which a sense of the importance of the duty assigned them imposed. They feel free to say that some of these Essays possess undoubted merit, both in matter and style, and they admit in them evidence of high scientific attainment as well as a familiarity with the graces of composition. But whilst cheerfully according these claims to their authors, the committee have preferred to be governed in their choice by considerations of originality and practical import, rather than of mere theoretic speculation, however finely portrayed. The committee, have, consequently, concluded to award but a single prize. The Essay selected is entitled "An Essay on a new method of treating Ununited Fractures and certain Deformities of the Osseous System." It bears a motto in French, (from Ambrose Paré,) which being literally rendered in modern English, reads, "and notwithstanding all the pains I have heretofore taken, I have reason to praise God, in that it hath pleased Him to call me to that branch of Medical practice commonly called Surgery, which can neither be bought by gold nor silver, but by industry alone and long experience."

If it please the Association I will now break the seal of the packet superscribed by the same motto, and declare the name of the successful competitor.

Dr. POPE then broke the seal and announced the name of Professor BRAINARD, of Chicago.

Dr. MCPHEETERS moved that Professor DANIEL BRAINARD be requested to take the stand and give the Association an abstract of his new mode of treating fractures, &c., which motion was carried, and Professor Brainard accordingly came forward, and gave the abstract desired.

Dr. HOOKER, of Connecticut, Treasurer, now introduced the subject of annual assessments, and called the attention of the Association to the fact that he was ready to receive the dues of members.

Dr. ELBERT, of Iowa, offered certain resolutions to the effect that a committee be appointed to recommend to the next annual meeting, for consideration, any alteration they may deem necessary in the Constitution, providing particularly that the election of officers, and the place of holding future annual meetings of the Association be determined by ballot, without the intervention of the Nominating Committee. The resolutions, after much discussion, were lost.

Dr. GUTHRIE offered the following resolutions, which were unanimously carried:

*Resolved,* That in the Secretary of the Treasury's recommendation to abolish or materially modify the duty on such crude drugs not produceable in this country, as are used in the laboratories of the country in the manufacture of chemicals, we recognise a wise provision for the further protection of the profession and the community at large from impure and sophisticated medicines.

*Resolved,* That a copy of this resolution be signed by the proper officers of this Association, and transmitted to the Secretary of the Treasury and to the Committee on Ways and Means.

Dr. J. B. JOHNSON, of St. Louis, asked and obtained permission to read the following letter from Dr. STEPHEN W. WILLIAMS, of Illinois:

*American Medical Biography, for the American Medical Association assembled at St. Louis.*

Mr. PRESIDENT: At the last meeting of the Association at New York, I presented the following preamble and resolution through my friend Dr. STEWART, of New York, which, with some amendments were laid on the table:

"As we are constantly called upon to deplore the ravages of death among the illustrious and worthy members of our profession throughout the United States,

*Resolved,* That a standing committee be appointed by this Association to procure memorials of the eminent and worthy dead among the distinguished physicians of our country, and present them to the Association for publication in the transaction.

I now beg leave to call up the resolution through my friend Dr. J. B. JOHNSON, of St. Louis. The medical biography of our country is intimately related to the history of it, as the lives of eminent men are identified with the history of the times in which they lived. In the United States there have been, and are to be found, medical men whose lives and actions are ornaments to human nature, and whose brilliant career in the cause of humanity and science reflect honor and dignity upon our country. We differ nothing in this respect in comparison with the learned and eminent physicians of Europe.

The profession of medicine contains more learned and distinguished men than any other profession or calling, and some memorial of their lives and actions should be presented to the world, in a more durable form than the periodicals of the day, and particularly of the newspaper press. A more permanent and proper place for the publication of such memorials, would be in the Transactions of the American Medical Association, and without disparagement to any other articles which have heretofore been published in these transactions—which memorials would be read by the members of the profession with great interest and improvement. Short biographies need take up but little room in the publication, and this objection to the proposed movement may be obviated. We are constantly noticing that death spares no rank or condition of men. Those who contend most skilfully against his insatiate ravages, themselves fall victims to his all conquering sword. Within a very short space of time, we have been called to lament the death of Dr. Nathaniel Chapman, the former President of this Association, of Drs. Samuel G. Morton, Wm. E. Horner, Isaac Parrish, G. S. Pattison, J. Kearney Rogers, Daniel Drake, the great medical pioneer of the West, Samuel McClellan, Amos Switchell, Abel Pierson, G. C. Shattuck, Archibald Welch, and many others which time will not permit us to enumerate.

This is not the place to speak their eulogies. Some permanent notice of them and many others who have recently died, should be published in the Transactions of this Association, where the useful improvements and discoveries of the living should be recorded, and the memories of the worthy dead should be preserved. I hope the resolution will prevail. [Signed,] STEPHEN W. WILLIAMS,
*Lavina, Winnebago county, Illinois, late of Deerfield, Mass.*

The Preamble and Resolutions referred to in the foregoing, were then on motion adopted. The Chair then announced that he would appoint the Committee contemplated by the Resolution hereafter.

Dr. McILVAINE offered the following resolution :

*Resolved,* That in the opinion of this Association, the practice of Professors reading lectures to their classes, no matter with how much care selected from the musty records of antiquity, is a miserable apology for teaching, is prima facie evidence of their inaptness to instruct, and is inimical to medical progress.

It was, on motion, laid on the table.

Dr. FRENCH submitted the following resolution, which was carried :

*Resolved,* That a Committee be appointed to enquire what State or other Society, represented in this Association, are in fellowship with irregular practitioners.

Dr. BLACHFORD of Troy, read a letter from Dr. A. D. SPORE, stating that he (Dr. SPORE) had been for some time investigating the subject of Hydrophobia, to ascertain what influence the weather had upon the disease, and in the letter he requested that communications on the subject might be sent to him by members of the Profession who had opportunities of making observations. Dr. SPORE not being a Delegate, it was moved that Dr. BLACHFORD be appointed Chairman of a Committee for the investigation of this subject.

Dr. McDOWELL, of St. Louis, offered the following resolution :

That a committee be appointed to investigate the improvements in the instruments for Lithotomy by Drs. NATHAN R. SMITH, PAUL F. EVE, and McDOWELL.

Dr. McDOWELL stated that his object in the appointment of this committee was, to settle the question as to the claims of the surgeons named to priority of invention in the field alluded to.

Dr. REYBURN, of St. Louis, moved that the investigation be postponed for a year, as Dr. N. R. SMITH, of Baltimore, one of the parties involved, was absent.

On motion, the whole subject was laid on the table.

On motion of Dr. SMITH, of Columbus, Ohio,

*Resolved,* That a Standing Committee of ——— be appointed by the Association on the subject of Insanity as it prevails in this country, including its *causes* as hereditary transmission, educational influences, physical and moral, social and political institutions, &c.; *its forms* and *complications, curability* and means of cure and prevention.

Adjourned to 3 P. M.

*Afternoon Session.*

The Association met as per adjournment.

Dr. White, of Buffalo, submitted the following resolution, which was carried:

*Resolved,* That the thanks of this Association be presented to Dr. J. Knight, late President, for the very dignified, courteous and efficient manner in which he presided over its deliberations, and that he be respectfully requested to furnish the usual address for publication.

The Committee appointed by the American Medical Association to devise or consider some comprehensive plan for the more general, systematic and thorough investigation of subjects connected with medical science, made a report, to which was appended the following resolution:

*Resolved,* That the American Medical Association hereby recommends all Medical Societies to establish, in accordance with the plan detailed in the foregoing report, special committees for the selection, investigation, collaboration and publication of all subjects of interest connected with medical science.

The resolution was carried, and the report and resolution was referred to the Committee on Publication.

Dr. Atlee communicated to the Association that he had received a letter from Dr. Parrish, Chairman of the Committee on Epidemics of New Jersey, stating that his report was yet unfinished, but would soon be ready for publication.

On motion, it was directed to be handed over to the Committee of Publication when finished.

Dr. N. S. Davis exhibited some samples of preserved milk, and asked the attention of the Association to the subject.

Adjourned to 9 A. M. to-morrow.

*Third Day—Thursday, 4th May.*

The Association convened at nine o'clock, A. M., Dr. Pope, President, in the Chair.

On motion, the regular order of business was suspended for the purposes of filling a vacancy in the Nominating Committee from Iowa.

On motion, Dr. McGuigan was chosen to fill the vacancy.

The minutes of Wednesday's proceedings were read, and after a few unimportant amendments, were adopted.

Dr. Atlee offered the following resolution, which carried:

*Resolved,* That it shall be the duty of the Publication Committee to append to each volume of the transactions hereafter published, a copy of the Constitution of the Association.

The following resolution, offered by Dr. GROSS, was also carried, and Dr. GROSS was appointed by the Chair the committee designated:

*Resolved,* That a committee of one be appointed by the Chair to inquire into the causes which obstruct the formation and establishment of our National Medical Literature, and to report the subject at the next annual meeting of this Association, or as soon thereafter as practicable.

Dr. J. BERRIEN LINDSLEY offered the following resolution, which, on motion, was referred to the Committee on Medical Education, with instructions to report at the next Annual Meeting of the Association :

*Resolved,* That this Association earnestly recommend to the few western schools which still retain the rule of making four years' practice equivalent to one term at college, the abrogation of said rule, as holding out a strong inducement and temptation to young men to enter upon the practice of medicine with little or no preparation.

Dr. PAUL F. EVE, of Nashville, Tenn., submitted the following resolution, which was carried :

*Resolved,* That a committee of three be appointed by the Chair, to report at the next meeting of the Association, the best means for preventing the introduction of disease by emigrants into our country.

The Chair appointed Drs. Dickson, Griscom and E. D. Fenner, this committee.

Dr. LINTON, of St. Louis, offered the following, which was also referred to the above named committee.

*Resolved,* That in the opinion of this Association, quarantine establishments afford no protection to States and cities against the invasion of epidemics such as cholera and yellow fever.

Dr. PENN offered a resolution to the following effect:

*Resolved,* That the members of the Committee of Arrangements who are not members of the Medical Association, be invited to take seats in this Association, as members by invitation.

Which was carried.

Dr. SAYRE, of N. York, called the attention of the Association to the offensive language of the Memorial of the American Medical Society of Paris, to the Association, which had been referred at a previous meeting, to the Committee on Education. Dr. SAYRE moved, that the memorial be withdrawn from the Committee on Education and laid on the table.

After an animated discussion, in which Drs. SAYRE, MCILVAINE, ATLEE, ELBERT, of Iowa, EDGAR and others participated, Dr. SAYRE'S resolution was carried.

On motion the regular business was suspended, to give place to the reading of the report of the Nominating Committee, of which the following is a copy :

*Report of the Committee on Nominations.*

The Committee on Nominations, in fulfilling the duty imposed upon them, recommend the continuance of several of the special committees previously created, and the appointment of some new ones. They, therefore, submit the following list of Chairmen of special committees, with the subjects to them committed:

Dr. Worthington Hooker, of New Haven, Connecticut—On epidemics of New England and New York.

Dr. John L. Atlee, of Lancaster, Pa.—On epidemics of New Jersey, Pennsylvania, Delaware and Maryland.

Dr. D. J. Cain, of Charleston, S. C.—On epidemics of South Carolina, Florida, Georgia and Alabama.

Dr. W. L. Sutton, of Georgetown, Ky.—On epidemics of Tennessee and Kentucky.

Dr. Thos. Reyburn, of St. Louis, Mo.—On epidemics of Missouri, Illinois, Iowa and Wisconsin.

Dr. Geo. Mendenhall, of Cincinnati, Ohio—On epidemics of Ohio, Indiana, and Michigan.

Dr. E. D Fenner, of New Orleans, La.—On epidemics of Mississippi, Louisiana, Arkansas and Texas.

Dr. James Jones, of New Orleans, La.—On the mutual relations of yellow and bilious remittent fever.

Dr. D. F. Condie, of Philadelphia, Pa.—On the causes of Tuberculous Disease.

Dr. Jos. Leidy, of Philadelphia, Pa.—On diseases of Parasitic Origin.

Dr. A. P. Merrill, of Memphis, Tenn.—On the Physiological Peculiarities and Diseases of Negroes.

Dr. Jos. N. McDowell, of St. Louis, Mo.—On statistics of the operation for the Removal of Stone in the Bladder.

Dr. F. P. Porcher, of Charleston, S. C.—On the Toxicological and Medicinal Properties of our Cryptogamic Plants.

Dr. Daniel Brainard, of Chicago, Ill.—On the Constitutional and Local Treatment of Carcinoma.

Dr. Geo. Engleman, of St. Louis, Mo.—On the Influence of Geological Formations on the Character of Disease.

Dr. Henry Taylor, of Mount Clemens, Mich.—On Dysentery.

Dr. Horace Green, of New York—On the use and effect of applications of Nitrate of Silver to the Throat in Local or General Disease.

Dr. P. C. Gooch, of Richmond, Va.—On the administration of Anaesthetic Agents, during Parturition.

Dr. Chas. Hooker, of New Haven, Conn.—On the Diet of the Sick.

Dr. E. R. Dabney, of Clarksville, Tenn.—On certain forms of Eruptive Fevers, prevalent in Middle Tennessee.

Dr. Sanford B. Hunt, of New York,—On the Hygrometrical State of the Atmosphere in various localities, and its influences on health.

Dr. Frank H. Hamilton, of Buffalo, New York,—On the frequency of deformities in fractures.

Dr. M. M. Pallen, of St. Louis, Mo.—On Diseases of the Prostate Gland.

Dr. H. A. Johnson, of Chicago, Ill.—On the Excretions as an index to the Organic Changes going on in the system.

Dr. Leroy H. Anderson, of Sumpterville, Ala.—On Typhoid Fever and its complications as it prevails in Alabama.

Dr. W. H. Byford, of Evansville, Ia.—On the Pathology and Treatment of Scrofula.

Dr. N. S. Davis, of Chicago, Ill.—On the Nutritive Qualities of Milk, and the influence produced thereon by pregnancy and menstruation in the human female, and by pregnancy in the cow, and also on the question whether there is not some mode by which the nutritive constituents of milk can be preserved in their purity and sweetness, and furnished to the inhabitants of cities in such quantities as to supersede the present defective and often unwholesome method of supply.

Dr. E. B. Haskens, of Clarksville, Tenn.—On the Microscopical Investigations of Malignant Tumors.

Dr. Geo. R. Grant, of Memphis, Tenn.—On the Sulphate of Quinia as a Remedial Agent in the Treatment of Fevers.

Dr. R. R. McIlvaine of Cincinnati, Ohio,—On the Study of Pathology at the Bedside.

Dr. E. S. Cooper, of Peoria, Ill.—On Orthopædic Surgery.

Dr. Andrew F. Jester, of Palmyra, Mo.—On the Modus Operandi of the Envenomed secretions of healthy Animals.

Dr. Saml. M. Smith, of Columbus, Ohio,—On Insanity.

Dr. Rene La Roche, of Philadelphia, Penn.—On the Jaundice of Yellow Fever in its Diagnostical and Prognostical Relations.

Dr. Charles Chandler of Rocheport, Mo.—On Malignant Periodic Fevers.

Dr. S. B. Chase, of Portland, Maine,—On Typhoid Fever in Maine.

Committee on Plans of Organization for State and County Societies—A. B. Palmer, M. D., Michigan; R. R. McIlvaine, M. D., Ohio; D. L. McGugin, M. D., Iowa; E. R. Peaslee, M. D., New Hampshire; Thos. Lipscomb, M. D., Tennessee.

Committee on Medical Literature—Robert J. Breckenridge, M. D., Kentucky, A. A. Gould, M. D., Massachusetts; D. L. McGugin, M. D., Iowa; J. B. Flint, M. D., Kentucky; O. M. Langdon, M. D., Ohio.

Committee on Medical Education—Wm. H. Anderson, M. D., Alabama; A. Lopez, M. D., do.; Andrew Murray, M. D., Michigan; F. A. Ramsay, M. D., Tennessee; R. D. Ross, M. D.

Committee on Prize Essays—Rene La Roche, M. D., Pennsylvania; Isaac Hays, M. D., do.; Alfred Stille, M. D., do.; J. B. Biddle, M. D., do.; Geo. W. Norris, M. D., do.; Joseph Carson, M. D., do.; Joseph Leidy, M. D., do.

Committee of Arrangements—Isaac Hays, M. D., Pennsylvania; G. Emerson, M. D. do.; Wilson Jewell, M. D., do.; Alfred Stille, M. D., do.; Francis West, M. D., do.; Wm. V. Keating, M. D., do.

*Committee of Publication*—Pliny Earle, M. D., New York; D. Francis Condie, M. D., Pennsylvania; E. S. Lemoine, M. D., Missouri; A. March, M. D., New York; E. H. Davis, M. D., do.; C. R. Gilman, M. D., do.; F. West, M. D., Pennsylvania.

After the reading of the report, Dr. Reyburn moved its adoption, excepting that portion referring to the Committee of Publication, in the following resolution:

*Resolved*, That the said report be adopted, with the exception of that portion which refers to the Committee of Publication.

A protracted and animated debate followed upon Dr. REYBURN'S resolution, which our limits do not allow us to give in extenso.

Dr. REYBURN objected to the Committee of Publication reported by the Nominating Committee, as its object was, obviously, to remove the publication of the Transactions from Philadelphia to New York. He deprecated change, as discourteous to the former Chairman, Dr. CONDIE, and he had no reason to anticipate that the Transactions could be issued in better style in New York than in Philadelphia.

Dr. SAYRE, of N. Y., thought that it would be unprecedented in the Annals of the Association, if any part of the report of the Nominating Committee should be rejected. He considered that the Association might as well appoint a permanent President as a permanent Committee of Publication. He had no fault to find with the former Committee, but he desired not to entail the labor and responsibilities of publication permanently upon any set of gentlemen.

Dr. STORER, of Boston, Massachusetts, made an earnest appeal against change in the place of publication. He was opposed to displacing the old Committee, because it was admitted that they had faithfully fulfilled their duties. He felt that to supersede them was a slight, and he was opposed to change, merely to gratify New York at the expense of Philadelphia.

Dr. PAUL F. EVE, of Tennessee, advocated the confirmation of the report as presented by the Nominating Committee. He could see no good reason for going behind it. Dr. EVE, moreover, was of opinion that too much space had been given in the last volume of the Transactions to a paper by a gentleman of Philadelphia. He had understood that $1000 or $1200 had been consumed in getting up this paper, and that the plates prepared at the expense of the Association had afterwards been sold for a trifle to a publishing house in Philadelphia, who issued the paper in separate form, thus interfering with the sale of the Transactions.

Dr. LEMOINE, of Missouri, thought that it had been understood at the last meeting, that the expense of the paper in question would not be objected to.

The debate was continued by Drs. PADDOCK, of Illinois, and HERRICK, of Illinois, in favor of the report of the Nominating Committee, and by Dr. MCPHEETERS, of Mo., in favor of Dr. REYBURN'S amendment.

A motion now prevailed, that the Association go into Committee of the Whole, on the subject, and Dr. ELBERT, of Iowa, was called to the Chair.

Dr. N. S. DAVIS, of Illinois, made a lengthy argument in favor of sustaining the Report of the Nominating Committee.

On the question being taken, the amendment of Dr. REYBURN was carried, and the Committee then rose, and the Association adjourned.

*Afternoon Session.*

The Association convened at three o'clock, Dr. POPE in the Chair.

A resolution was offered by Dr. STORER in relation to permanent members, which was unanimously carried.

A resolution was offered to the effect that W. S. MAUS, M. D., of Pekin, Illinois, be elected a permanent member—which was carried unanimously.

Dr. ATLEE offered the following resolution, which was carried.

*Resolved*, That this Association earnestly recommend to their medical brethren in those States in which Societies do not exist, the immediate organization of State and County Medical Societies.

Dr. RAMSEY offered a resolution, which, on motion of Dr. COONS, was laid on the table.

Dr. BRECKENRIDGE offered a resolution to the following effect:

*Resolved*, That the papers and documents of the Association shall hereafter be the exclusive property of the Association. Carried.

Dr. PHELPS, of New York, requested and obtained leave to read a document which he presented in the morning, and the reading of which had been deferred to allow the Committee on Nominations to make their report.

"The document purported to be an abstract of a paper which traces the connexion existing between medicine and religion in its origin and progress."

The document was referred to a Special Committee, to be appointed by the Chair. The President named Dr. ATLEE, Dr. SAYRE, and Dr. MARCH, as the Committee.

A motion was now made to proceed to the regular business, which was carried.

Dr. EVE moved that in the matter relating to the report of the Committee on Nominations, the blank occasioned by the amendment offered by Dr. REYBURN, which was adopted in Committee of the Whole, be referred back to the Nominating Committee for the purpose of filling up the blank, which was lost.

Dr. BRODIE now moved that the blank made by striking out the names of the Publication Committee in Committee of the Whole, be filled by re-inserting the names as reported by the Nominating Committee. This motion was advocated at length by Drs. BRECKENRIDGE, of Ky.; SAYRE, of N. Y.; McDOWELL, of Mo.; WHITE, of N. Y.; PALMER, of Mich.; McILVAINE, of Ohio, and others; and was opposed by Drs. ATLEE, of Pa., and STORER, of Mass. It finally prevailed, thus settling the question in favor of the report originally made by the Nominating Committee, which transfers the Publication of the Transactions of this meeting from Philadelphia to New York.

After this vote was announced, Dr. LA ROCHE, of Philadelphia, on behalf of the Philadelphia delegation, tendered the resignation of Dr. CONDIE, as Treasurer, which was accepted.

On motion of Dr. BIDDLE, Dr. CHARLES HOOKER was elected Treasurer. Dr. HOOKER, however declined to accept the office.

Dr. WEST, of Philadelphia, one of the Secretaries, then tendered his resignation, and the question being upon accepting it, it was lost.

Dr. BRECKENRIDGE, of Kentucky, then offered the following resolution, which was seconded by Dr. Storer, of Mass., and carried :

*Resolved*, That hereafter the majority of the Committee of Publications shall be selected from the physicians of that city in which the Association may annually meet.

A vote of thanks was then unanimously returned to Dr. CONDIE for the able, zealous and impartial manner with which he had discharged his duties as Treasurer.

A resolution was offered to amend the constitution, by providing that its annual meetings shall be held on the second Monday in May, instead of the first Monday. The resolution, under the rule, lies over for a year.

Dr. W. H. BYFORD offered a resolution of thanks to Dr. PINCKNEY, of the United States Navy, for the able address delivered before the Association on the first day of its session, which was unanimously adopted.

Dr. ATLEE offered a resolution tendering the thanks of the Association, and of the individual members, to the citizens of St. Louis for their hospitality and kindness ; also, to the Directors of the various railroads and officers of steamboats, for the generous manner in which they have tendered their kind offices, which was adopted.

Dr. WHITE moved that a vote of thanks be extended to the late Publishing Committee for their faithful endeavors to serve the Association, which was unanimously adopted.

A resolution offered by Dr. W. W. HITT, touching alcoholic drinks, was referred to the Committee on Nominations.

A resolution offered by Dr. C. B. HUGHES, relating to specialty practice in Surgery, was on motion laid on the table.

Dr. WHITE, from the Nominating Committee, reported to the Association the name of Dr. BLATCHFORD, of New York, as Treasurer, in the place of Dr. CONDIE, resigned.

Dr. BLATCHFORD declined acting in that capacity, and the committee subsequently reported the name of Dr. ISAAC WOOD, of New York. They also reported a special committee on Epidemics, for the States of Virginia and North Carolina, with Dr. HASKINS as Chairman; and also, the resolution relative to alcoholic drinks was reported back by them, referring it to a special committee, consisting of Dr. MUSSEY.

Dr. W. S. EDGAR offered a resolution in regard to the compounding of medicines, and recommending apothecaries to use different colored paper in putting up poisonous drugs, with an appropriate stamp upon it, in contradistinction to other medicines.

On motion of Dr. SMITH, of N. J., Dr. McDOWELL's resolution, asking for a committee of investigation on claims to priority of invention of lithotomy instruments, was taken up ; and on motion of Dr. McDOWELL, who said he did not wish to press it in the absence of Dr. N. R. SMITH, it was laid over to next year.

Dr. BANE, of Illinois, was elected a permanent member.

A letter from Dr. ENGELMAN, of St. Louis, was read, resigning his situation as Chairman on one of the special committees, but the Association refused to accept it.

The following resolution, offered by Dr. J. B. LINDSEY, was, on motion, laid on the table :

*Resolved,* That the too prevalent practice of Professors in Medical Colleges recommending their own writings and editings as text books for their students, is, in the opinion of the Association, a serious evil, trammelling as it does, the student in his choice of books, and promoting the publication and circulation of works of inferior merit.

A vote of thanks was returned to Dr. HOOKER, Treasurer pro tem.

Dr. GROSS informed the Association that the second volume of the work of the late Professor DRAKE, of Cincinnati, was now in the press, at Philadelphia, and would be issued early in the present summer. The second volume he said was on Practical Medicine, and will be entirely independent of the first,

The Association then adjourned *sine die.*

